# Circular RNAs in diabetes and its complications: Current knowledge and future prospects

**DOI:** 10.3389/fgene.2022.1006307

**Published:** 2022-10-26

**Authors:** Wenfeng Yin, Ziwei Zhang, Zilin Xiao, Xia Li, Shuoming Luo, Zhiguang Zhou

**Affiliations:** National Clinical Research Center for Metabolic Diseases, Key Laboratory of Diabetes Immunology (Central South University), Ministry of Education, and Department of Metabolism and Endocrinology, The Second Xiangya Hospital of Central South University, Changsha, China

**Keywords:** circular RNAs, biomarkers, type 1 diabetes, diabetes, diabetic complication

## Abstract

A novel class of non-coding RNA transcripts called circular RNAs (circRNAs) have been the subject of significant recent studies. Accumulating evidence points that circRNAs play an important role in the cellular processes, inflammatory expression, and immune responses through sponging miRNA, binding, or translating in proteins. Studies have found that circRNAs are involved in the physiologic and pathologic processes of diabetes. There has been an increased focus on the relevance of between abnormal circRNA expression and the development and progression of various types of diabetes and diabetes-related diseases. These circRNAs not only serve as promising diagnostic and prognostic molecular biomarkers, but also have important biological roles in islet cells, diabetes, and its complications. In addition, many circRNA signaling pathways have been found to regulate the occurrence and development of diabetes. Here we comprehensively review and discuss recent advances in our understanding of the physiologic function and regulatory mechanisms of circRNAs on pancreatic islet cells, different subtypes in diabetes, and diabetic complications.

## Introduction

Diabetes is a major disease that threatens global health. It is spiraling out of control and 537 million adults aged 20–79 years are living with diabetes around the world in 2021 according to the International Diabetes Federation Diabetes Atlas 10th edition. This represented 9.8% of the world’s population in this age group (International Diabetes Federation, 2019). It is responsible for 6.7 million deaths and has become a major global public health problem. Diabetes can be classified into the following general categories: type 1 diabetes (T1D), type 2 diabetes (T2D), gestational diabetes mellitus (GDM), and diabetes due to other causes (American Diabetes Association, 2020). Poor glycemic control, late diagnosis of diabetes, and alterations of inflammatory cytokines are key pathologic processes that cause impaired cellular and organ functions, contributing to a series of chronic complications that include diabetic retinopathy (DR), diabetic nephropathy (DN), diabetic neuropathy (DNP), diabetic cardiomyopathy (DCM) and hyperglycemia- related endothelial dysfunction and wound healing. These cause a high rate of disability and mortality, and represent a heavy economic burden on society and families. A complete cure for diabetes and its complications has not yet been found because of complicated pathogenesis, which means that patients need to receive treatment for the rest of their lives.

Although great efforts have been made to reveal the mechanisms underlying the pathogenesis of diabetes and its complications, its exact mechanisms remain largely unclear. The main pathophysiologic change in diabetics is β cell dysfunction and failure, which is closely related to the expression of encoded proteins and non-coding transcript changes. Mounting evidence suggests that different types of non-coding RNA, including microRNAs (miRNAs) and circRNAs, are key players in the regulation of β cell function and diabetes (Latreille et al., 2014; Wong et al., 2018).

CircRNAs are a new class of non-coding RNAs that differ from traditional linear RNAs. Thousands of human circRNAs were recently identified using molecular biology strategies coupled with new bioinformatics approaches. Existing studies have shown that circRNAs play a very important role in several human diseases, including tumors (Lu et al., 2017; Zhang et al., 2018), neurologic diseases (Kumar et al., 2017; Salta and De Strooper, 2017), immune diseases (Li et al., 2016; Iparraguirre et al., 2017), and cardiovascular diseases (Vausort et al., 2016; Wang et al., 2016), and accumulate during aging (Westholm et al., 2014; Cortes-Lopez et al., 2018). An increasing number of studies have attempted to identify new circRNAs and their potential biologic functions in diabetes and its related complications. CircRNAs not only are the molecular markers of disease diagnosis, but also are key regulators of the occurrence and development of diseases that affect disease prognosis and outcome (Salzman, 2016; Han et al., 2017). This paper comprehensively reviews and discusses recent advances in our understanding of the physiologic function and regulatory mechanisms of circRNAs in different subtypes of diabetes and its complications.

## Characteristics and biological functions of circRNAs

CircRNAs are produced by a non-canonical splicing event called back splicing and are widely present in eukaryotic cells (Kristensen et al., 2019). CircRNAs can be classified into three types by their production by different regions of genes: exonic circRNAs, intronic circRNAs, and exon-intron circRNAs (Ghasemi et al., 2019). Exonic circRNAs are the most common form of circRNAs that involve approximately 80% of the total circRNAs and are mainly located in the cytoplasm (Salzman et al., 2012; Memczak et al., 2013). They are expressed from known protein-coding genes and consist of one or more exons (Guo et al., 2014), whose 5′ and 3′ ends are covalently linked to form a ‘head-to-tail’ splice junction (Meng et al., 2017). The expression levels of circRNAs and their corresponding linear mRNAs do not always correlate with each other (Salzman et al., 2013).

CircRNAs have some conserved sequences. For instance, 457 of the 2121 circRNAs in humans were found to have circular orthologues in mice (Memczak et al., 2013), and approximately 4% of humans and mice orthologous genes can generate circRNAs (Salzman et al., 2013). CircRNAs exhibit high stability compared with linear RNAs (Jeck et al., 2013; Memczak et al., 2013). This high stability is because their covalently closed ring structure protects these molecules from exonuclease-mediated degradation (Jeck et al., 2013). CircRNAs are expressed across the eukaryotic tree of life, and comprise >10% of all transcripts in human cells (Salzman et al., 2012). The expression levels of circRNAs vary greatly, with time-space specificity and tissue-cell specificity.

**FIGURE 1:**
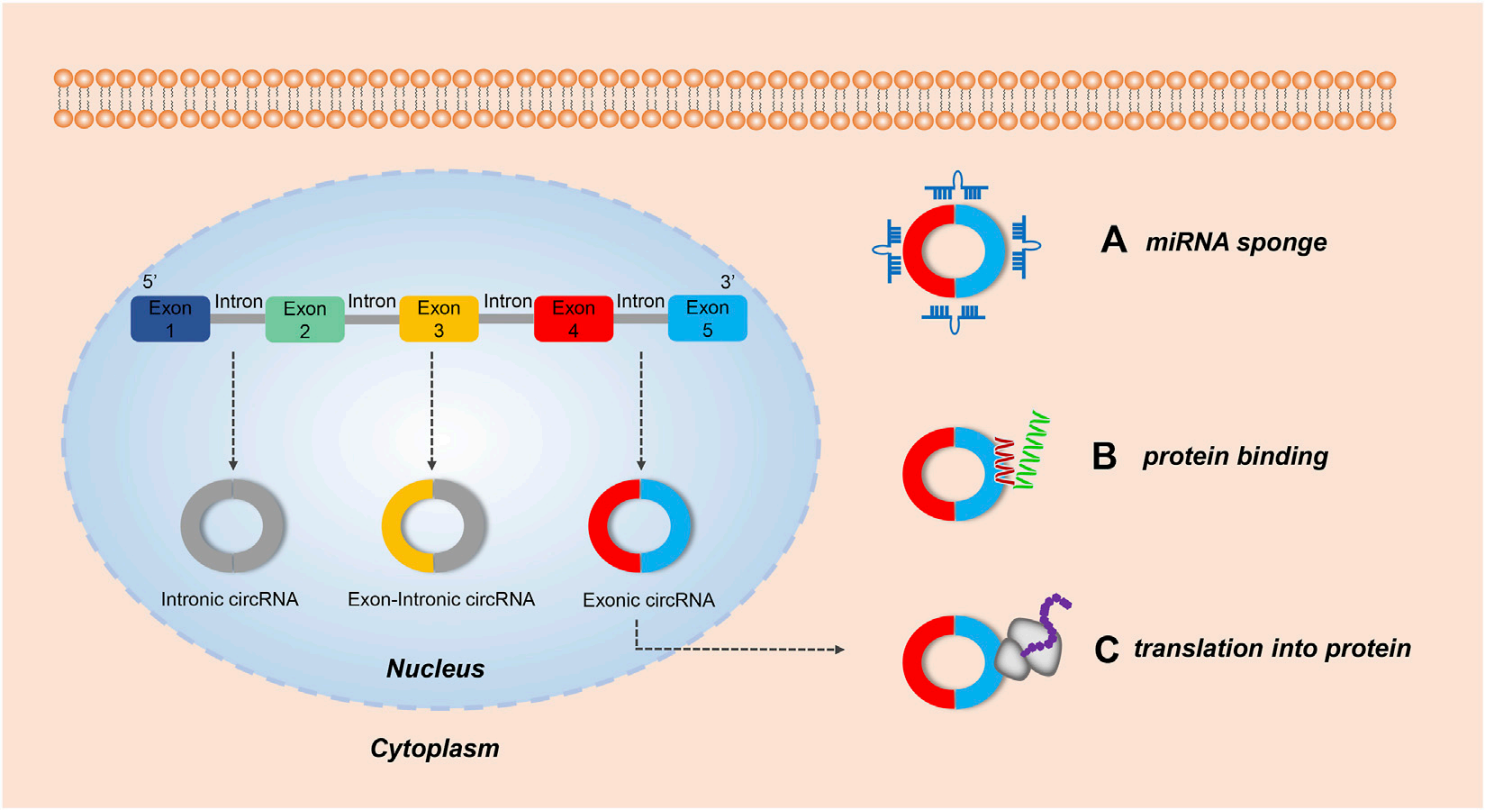
The biological functions of circRNAs. Both intronic circRNAs and exon-circRNAs play a role in the nucleus, while exonic circRNAs exhibit several roles in the cytoplasm. **(A)** Acting as a miRNA sponge: by adsorbing miRNA, some exonic circRNAs can regulate the function of miRNA-target mRNAs. **(B)** Serving as a protein sponge: several exonic circRNAs can interact with RNA-binding proteins (RBP) and mediate their action. **(C)** Translating protein: some exonic circRNAs modified with an internal ribosome entry site can encode protein.

The unique characteristics of circRNAs give them diverse biological functions and broad application prospects in medicine and the life sciences (Liu and Chen, 2022) (Figure 1). Most important is that circRNAs can regulate gene expression by acting as effective miRNA sponges (Memczak et al., 2013), which are more effective than linear RNAs in miRNA sponging (Ebert et al., 2007; Tay et al., 2015). In other words, circRNA can act as a stable and efficient miRNA inhibitor. As a classic example, the native circRNA CDR1as acts as a “sponge” for miR-7a/b in human and mouse brains (Hansen et al., 2013a). CDR1as contains 74 miR-7 binding sites and negatively regulates miR-7 (Hansen et al., 2013b). When CDR1as is overexpressed, it can bind to miR-7 in a large amount and lead to an increased expression level of the miR-7 target gene. While it is inhibited, the expression level of the miR-7 target gene is decreased (Salmena et al., 2011). Thus, the regulation mechanism of miRNA sponges in circRNA significantly affects the body’s biological function. As far as we know, the miRNA sponge regulation mechanism is the primary motivator for the study of the mechanism of circRNA action as it is of great significance for the diagnosis and treatment of diseases and potential drug development.

CircRNAs can act as protein sponges and stably regulate the activity of RNA-binding protein (RBP) (Ashwal-Fluss et al., 2014). They can be used as biological markers for disease detection (Iparraguirre et al., 2017; Zhang et al., 2018), and act as therapeutic agents and targets (Holdt et al., 2018). Finally, in a limited number of cases circRNAs can also be translated (Legnini et al., 2017; Yang et al., 2017; Yang et al., 2018; Lei et al., 2020). Further understanding of the biology of circRNAs offers great promise for the targeting of novel strategies against a wide spectrum of disease entities.

## Specifically expressed circRNAs in pancreatic islet cells

**FIGURE 2:**
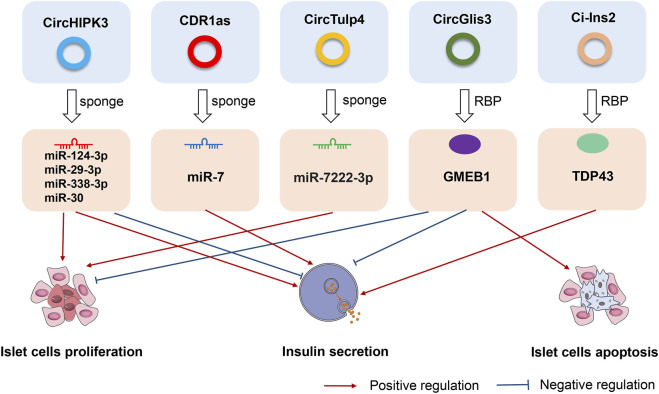
The roles and effects of circRNAs on pancreatic islet β cells. CircHIPK3, CDR1as, circTulp4, circGlis3, and ci-Ins2, are the most representative circRNAs in pancreatic islet β cells. CircHIPK3, CDR1as, and circTulp4 are miRNA sponges that affect cell proliferation, apoptosis, and insulin secretion. CircGlis3 suppresses the proliferation of islet β cells, inhibits insulin secretion, and promotes cell apoptosis by interacting with the RNA-binding protein glucocorticoid modulatory element-binding protein 1 (GMEB1). Ci-Ins2 can promote insulin secretion by interaction with the RNA-binding protein TAR DNA-binding protein 43 kDa (TDP43).

Deranged islet cell function is a key cause of both T1D and T2D. Recent studies have identified α- and β-cell-selective transcriptomic signatures in human islets (Dorrell et al., 2011; Ackermann et al., 2016). Specific regulatory elements such as non-coding RNAs have been identified as essential components for the maintenance of β-cell activity and function (Arnes et al., 2016; Mirza et al., 2017). Recent work found that circRNA, as a new member of the non-coding RNA family, might play similar roles in the maintenance of the β-cell identity and function. Stoll et al. (Stoll et al., 2018) detected ≈3,000 circRNAs in human islets using a microarray-based approach that covered only a fraction of the previously annotated circRNAs. Kaur and his colleagues detected ≈10,000 circRNAs in human α-, β-, and exocrine cells using published high-depth RNA-seq datasets (Kaur et al., 2018a). The most highly expressed circRNAs host genes were MAN1A2 and HIPK3, which both exhibit alternate circular isoforms in the three cell-types. In their analysis, 33 α cell-selective and 35 β cell-selective circRNAs were identified as highly and selectively expressed, including circTGFBR3, which is highly β cell-selective, and FAP, SYTL5, PTPRT, STK32B, and BVES, which are highly α cell-selective transcripts (Kaur et al., 2018a). Furthermore, differential expression analysis identified 14 downregulated and 22 upregulated circRNAs in β-cells compared with α-cells. The highly upregulated circRNAs in β-cells included circTGFBR3 and circHDAC9, while the highly downregulated circRNAs included circFAP and circGLS (Kaur et al., 2018a). These newly identified cell-selective circRNAs in human islets offer new avenues for exploring their functional significance in the maintenance of β-cell function. In Figure 2, we have listed several most representative circRNAs in pancreatic islet β cells. These circRNAs not only regulated the proliferation and apoptosis of islet β cells, but also were involved in the progression of different subtypes of diabetes and its complications. In the next sections, we specifically described and discussed the roles and effects of these circRNAs.

## CircRNAs and different types of diabetes

**FIGURE 3:**
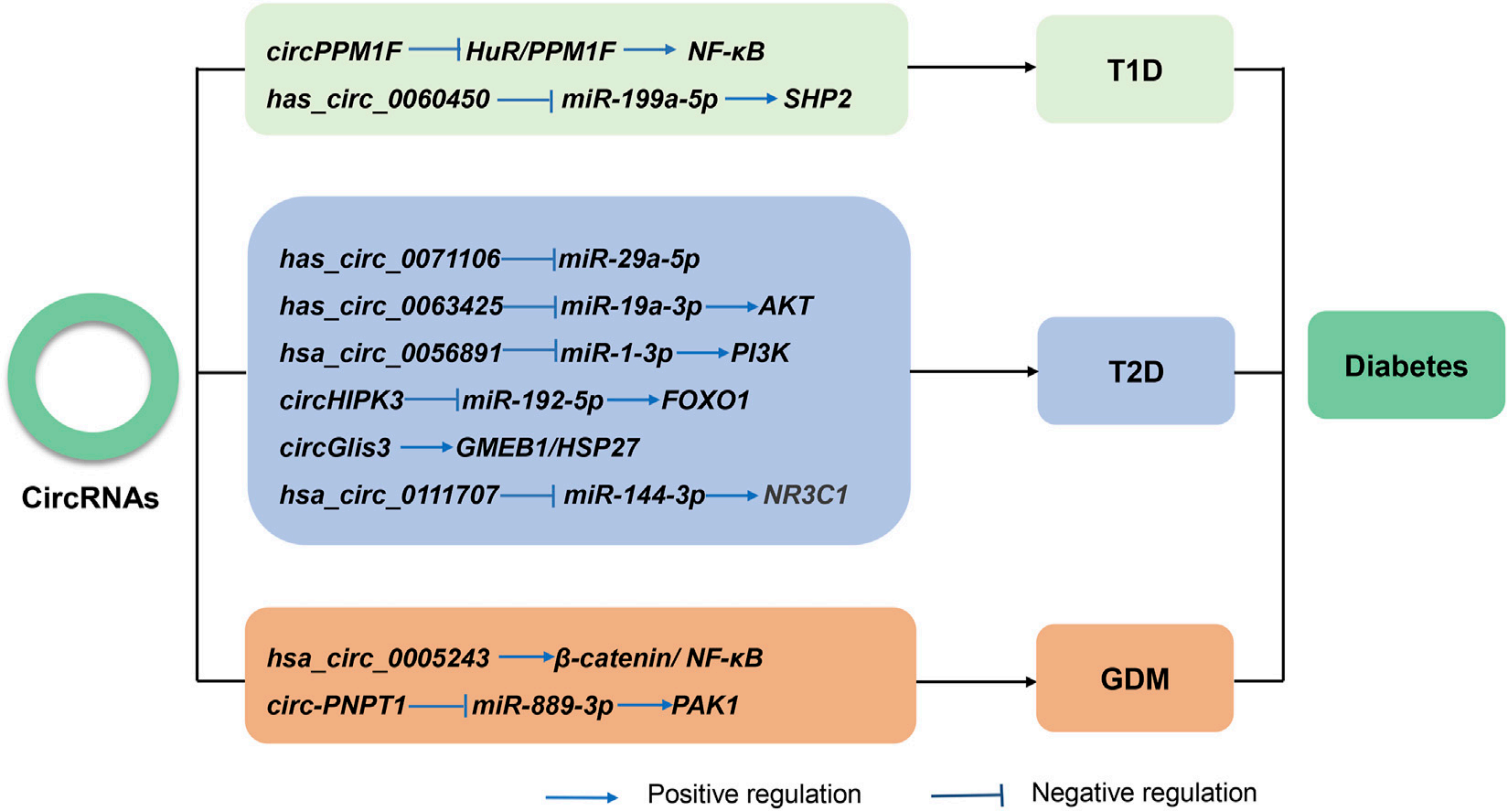
The known roles and regulatory pathways of circRNAs involved in type 1 diabetes (T1D), type 2 diabetes (T2D), and gestational diabetes mellitus (GDM).

Thus far, multiple studies have reported the abnormal expression of circRNAs in almost all diabetes types (Figure 3). Several works have also found that circRNAs regulate cellular dysfunction, immune inflammation, and autophagy. The following sections will discuss the latest research on circRNAs in different types of diabetes.

### Type 1 diabetes

T1D, also known as insulin-dependent diabetes, is a chronic autoimmune disease that is characterized by β-cell dysfunction and destruction, an absolute lack of insulin, and elevated blood glucose levels (Dayan et al., 2021). Substantial evidence has been published that describes the function of circRNAs in autoimmune disorders, which is in part due to the evaluation of autoimmune diseases in T1D research.

#### Biomarker circRNAs

A total of 61 upregulated and 7 downregulated circRNAs have been found in the plasma of patients with new-onset T1D, of which four circRNAs (hsa_circRNA_101062, hsa_circRNA_100332, hsa_circRNA_085129, and hsa_circRNA_103845) have been successfully validated with quantitative real-time reverse transcription-PCR (Li et al., 2020a). At the same time, 93 differentially expressed circular transcripts were identified in the peripheral blood of T1D patients compared with controls, of which 30 and 63 were up- and downregulated, respectively. GO and KEGG analysis suggest that these circRNAs may take part in the development of T1D *via* different pathways. Another, hsa_circ_0072697 was identified as a potential endogenous miRNA sponge for regulating β-cell function (Luo et al., 2020). These aberrantly expressed circRNAs might be biomarkers for T1D, and longitudinal studies are warranted to validate them.

#### Therapeutic circRNAs

CircRNAs are involved in inflammation response in the process of T1D and maybe provide a novel therapeutic target for T1D. Emerging evidence has suggested that circPPM1F was predominantly expressed in monocytes and promoted the activation of M1 macrophages through the circPPM1F-HuR-PPM1F-NF-*κ*B pathway. Overexpression of circPPM1F could contribute to pancreatic islet injury (Zhang et al., 2020a). Macrophages, inflammatory mediators of the immune response, play a prominent function in insulitis and promote inflammatory cell infiltration by supporting autoimmune T cells in the development of T1D (Cnop et al., 2005). Yang and coworkers found that has_circ_0060450 was upregulated in patients with T1D, and acted as a sponge that targets adsorbed miR-199a-5p to suppress macrophage-mediated inflammation by inhibiting the JAK-STAT signaling pathway (Yang et al., 2020). Additionally, 1922 markedly expression circRNAs were found in mouse islet β-cells induced by various cytokines, such as interleukin-1β (IL-1β), interferon-γ(INF-γ), and tumor necrosis factor-α (TNF-α) (Wang et al., 2021a). In order to clarify the role of circRNA in T1D, Yi et al. (Yi and Cheng, 2021) established the circRNA-lncRNA-miRNA-mRNA ceRNA regulatory network for T1D using computational and bioinformatic approaches. However, studies on therapeutic circRNAs in T1D are limited, it needs to be proven by a large amount of data in the future.

### Type 2 diabetes

The prevalence of T2D is rising due to increased living standards and altered diet structure, thereby creating a huge health burden for both patients and society. Recent studies have focused on exploring the effects of circRNAs on the pathogenesis and progression of T2D, reporting that circRNAs may be candidates for diagnostic biomarkers and therapeutic targets of diabetic patients.

#### Biomarker circRNAs

Serum hsa_circ_0054633 could diagnose pre-diabetes and T2D patients in a cohort comprising 60 control subjects, 63 pre-diabetes subjects and 64 T2D subjects (Zhao et al., 2017). What’s more, serum hsa_circ_0054633 levels in T2D patients were correlated with fasting blood glucose, hemoglobin A1c, and low-density lipoprotein (Liang et al., 2021a). Another, the combination of has_circ_0063425 and hsa_circ_0056891 could discriminate T2D and impaired fasting glucose patients from healthy controls in a cohort of 313 subjects. The areas under the curves (AUCs) were 0.837 and 0.719, respectively (Lu et al., 2021). More importantly, the study found has_circ_0063425 and hsa_circ_0056891 play a significant role in the insulin phosphatidylinositol 3-kinase/protein kinase B signaling pathway *via* sponging miR-19a-3p and miR-1-3p, respectively (Lu et al., 2021). Additionally, circHIPK3 and CDR1as were upregulated in T2D patients (Rezaeinejad et al., 2022). CircHIPK3, also named hsa_circ_0000284, is one of the most abundant circRNAs in pancreatic islets. It originates from exon 2 of HIPK3, which is very conserved between human cells and mice (Kaur et al., 2018a). It was verified that circHIPK3 contributes to hyperglycemia and insulin resistance by sponging miR-192-5p and up-regulating the expression levels of FOXO1 (Cai et al., 2020). Additionally, it has been reported that circHIPK3 is decreased in DB/DB islets. Depletion of circHIPK3 results in increased β-cell apoptosis and decreased glucose-stimulated insulin secretion. CircHIPK3 appears to have a vital effect on islet cells partly through the miRNA sponging of miR-124-3p, miR-29-3p, miR-338-3p, and miR-30 *via* regulation of the expression of key β-cell genes such as Slc2a2, Akt1, and Mtpn (Stoll et al., 2018). There is sufficient evidence that all these miRNAs have a significant role in β-cell development, proliferation, survival, or function (Baroukh et al., 2007; Joglekar et al., 2009; Bagge et al., 2012; Jacovetti et al., 2015). CircHIPK3 could distinguish T2D patients from healthy control subjects with a sensitivity of 50.0% and a specificity of 88.4% (Rezaeinejad et al., 2022). CDR1as originally described in brain tissue (Hansen et al., 2013a), is also enriched in pancreatic β-cells. CDR1as can regulate insulin transcription and secretion in islet cells *via* inhibition of miR-7 function by modulating the expression of the endogenous targeted genes Myrip and Pax6 (Xu et al., 2015). The level of CDR1as was decreased in both OB/OB and DB/DB mice, which is consistent with its involvement in diabetes pathogenesis. Over-expression of CDR1as upregulates islet cell insulin secretion and insulin content (Xu et al., 2015), and loss of CDR1as abrogates the beneficial effects of CDR1as upregulation on islet cells and decreases the prolactin-induced proliferation of MIN6B cells in isolated rat islets (Stoll et al., 2018). Study showed that CDR1as can identify prediabetic patients from healthy control subjects (AUC = 0.605) (Rezaeinejad et al., 2022).

#### Therapeutic circRNAs

Circ-Tulp4 might be a potential therapeutic intervention for T2D (Wu et al., 2020). *In vitro* experiments indicated that circTulp4 can regulate the proliferation of the MIN6 cell line. Moreover, upregulation of circTulp4 promoted the expression of SOAT1, resulting in increased cyclin D1 expression, thereby reinforcing cell cycle progression and reducing β-cell dysfunction *via* miR-7222-3p inhibition (Wu et al., 2020). CircGlis3, a β cell-derived exosomal circRNA was found to be elevated in the serum of diabetic mice and participants with T2D and it could be transferred to islet endothelial cells as a therapeutic target (Xiong et al., 2022). CircGlis3 is involved in lipotoxicity-induced β cell disorder and the development of diabetes by suppressing insulin secretion and cell proliferation. Mechanistically, circGlis3 reduces the viability and migration of islet endothelial cells and is delivered to target cells *via* exosomes. Importantly, intronic circRNAs generated from insulin genes also play a critical role in the development of T2D. For example, circular intronic-Ins2 (ci-Ins2) could regulate β-cell function (Das et al., 2020). Evidence indicated that circRNA containing the lariat sequence of the second intron of the insulin gene can regulate insulin secretion in rodent diabetes models and patients with T2D, possibly explaining their impaired secretory capacity (Stoll et al., 2020). There is an association between circANKRD36 and chronic inflammatory cytokines in T2D. Further functional experiments found that circANKRD36 levels are positively correlated with those of blood glucose and IL-6 (Fang et al., 2018). Additionally, in T2D rats, dysregulated expression circRNAs has been linked to the autophagy of pancreatic β-cells. It might be caused by rno_circRNA_008565 through interaction with miRNAs (Bai et al., 2019). All together, circRNAs may associate with insulin resistance, inflammatory response, and β-cell failure in T2D and serve as therapeutic targets for T2D in the future.

## Gestational diabetes mellitus

### Biomarker circRNAs

CircRNAs play key roles in predicting multiple adverse events in patients with GDM. CircVEGFC a novel regulator of glucose metabolism, is increased in pregnant women with GDM. High levels of circVEGF are associated with fetal malformations and hypertension (She et al., 2021). Pregnant women with high levels of circACTR2 expression are more likely to suffer from GDM. Zhu et al. (Zhu et al., 2021a) showed that abnormal circACTR2 expression is correlated with fetal malformations, premature delivery intrauterine infection, and miscarriage. These findings suggest that circRNAs can potentially serve as biomarkers for predicting the risk of complications in GDM patients. However, the clinical application of circRNAs and their underlying mechanisms require further study.

#### Therapeutic circRNAs

GDM is a common complication of pregnancy that poses a serious threat to the health of both the mother and child. The roles of therapeutic circRNAs in GDM have also been reported. For instance, the expression profiles of the placental villous circRNAs of in women with GDM have been described. These deregulated circRNAs might be associated with GDM (Yan et al., 2018; Tang et al., 2020a). Chen et al. (Chen et al., 2021) reported that circ_0008285 is highly expressed in GDM patients. Loss of circ_0008285 could suppress cell proliferation, invasion, and migration. In addition, hsa_circ_0005243 was implicated in reducing cell proliferation and migration, as well as increasing the secretion of TNF-α and IL-6 in GDM by targeting the β-catenin/NF-*κ*B axis (Wang et al., 2020). Another, circPNPT1 is increased in the placental tissues of GDM and high glucose (HG)-induced trophoblast cells (Zhang et al., 2021a). Mechanistically, circPNPT1 can regulate trophoblast cell dysfunction by sponging miR-889-3p and activating the expression of p21-activated kinase 1. More importantly, circPNPT can also be packaged into exosomes and transferred to other cells (Zhang et al., 2021a).

## The role of circRNAs in diabetic complications

**FIGURE 4:**
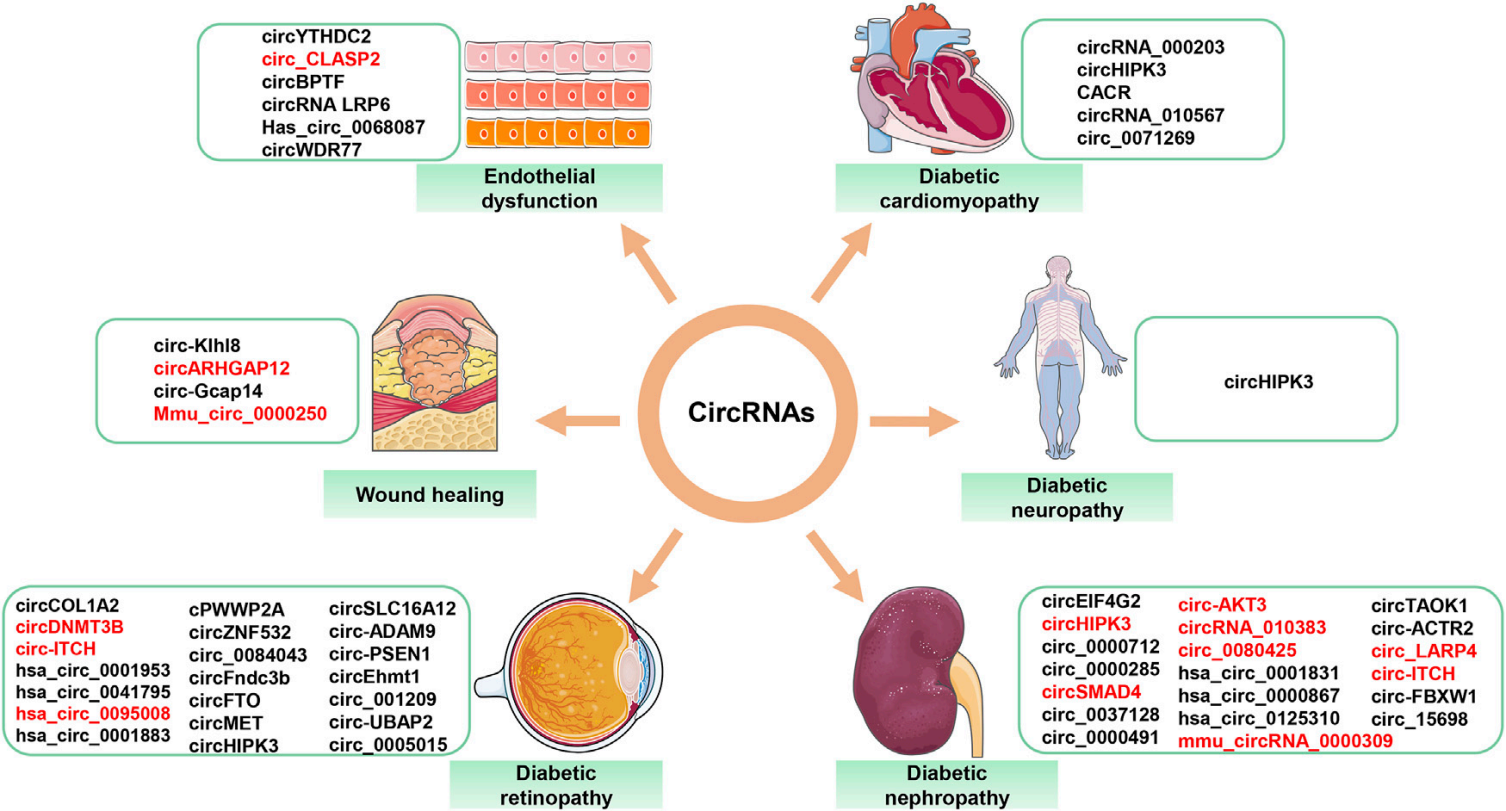
Aberrantly expressed circRNAs in diabetes-related diseases. Red font = decreased circRNAs; black font = increased circRNAs.

Persistent hyperglycemia leads to the development of diabetic complications, such as DR, DN, DNP, DCM, and high glucose-related endothelial dysfunction and wound healing. These complications pose a serious threat to human life. Numerous studies have demonstrated the importance of circRNAs in the development of diabetic complications. CircRNAs with confirmed roles in diabetes-related diseases are summarized (Figure 4). Table 1 describes in detail the roles of circRNAs in diabetic complications.

**TABLE 1:** The expression and roles of circRNAs in diabetic complications.

Disease	CircRNA name	Source	Expression	Target	Biological functions	Reference
DR	CircCOL1A2	Human retinal microvascular endothelial cells (HRMECs)	Up	MiR-29b/VEGF	Promoting angiogenesis	Zou et al. (2020)
	CircDNMT3B	HRMECs	Down	MiR-20b-5p/BAMBI	Reducing the number of retinal acellular capillaries and reducing visual damage	Zhu et al. (2019)
	Circ-ITCH	Diabetic retinal pigment epithelial cells in rat	Down	miR-22/MMP-2, MMP-9, TNF-*α*	Involved in the physiology and pathology of DR	Zhou et al. (2021)
	Circ_0005015	Plasma of DR patients	Up	MiR-519d-3p/MMP-2, XIAP, STAT3	Facilitating retinal endothelial angiogenic function	Zhang et al. (2017)
	Hsa_circ_0001953	Blood of proliferative diabetic retinopathy patients	Up	__	Serving as a novel diagnostic biomarker for PDR	Wu et al. (2021a)
	Hsa_circ_0041795	ARPE-19 cells	Up	MiR-646/VEGFC	Contributing to ARPE 19 cells injury	Sun and Kang, (2020)
	CircHIPK3	Retinal endothelial cell	Up	MiR-30a-3p/VEGFC, FZD4, WNT2	Increasing endothelial proliferation and vascular dysfunction	Shan et al. (2017)
	CPWWP2A	Vascular pericytes	Up	MiR-579/SIRT1	Affecting diabetes-induced microvascular dysfunction	Liu et al. (2019)
	CircZNF532	Serum of DR patients and ARPE-19 cells	Up	MiR-20b-5p/STAT3	Regulating the apoptosis and pyroptosis of ARPE-19 cells	Liang et al. (2021b)
	CircZNF532	Vascular pericytes	Up	MiR-29a-3p/NG2, LOXL2, CDK2	Coordinating pericyte biology and vascular homeostasis in DR	Jiang et al. (2020)
	CircZNF532	DR patients and (HRMECs)	Up	MiR-1243/CARM1	Facilitating angiogenesis and inflammation in DR	Wang et al. (2022b)
	Circ_0084043	ARPE-19 cells	Up	MiR-140-3p/TGFA	Contributing to the progression of DR	Li et al. (2020b)
	CircFndc3b	Serum of PDR patients	Up	__	Facilitating angiogenesis	Li et al. (2021)
	CircFTO	Vitreous humor of DR	Up	MiR-128-3p/TXNIP	Promoting angiogenesis and impairing the blood-retinal barrier	Guo et al. (2021)
	CircMET	Retinas of mice with DR	Up	IGF2BP2, NRARP/ESM1	Coordinating tip cell specialization and pathologic angiogenesis	Yao et al. (2022)
	CircSLC16A12	Serum of DR patients and HRMECs	Up	MiR-140-3p/FGF2	Inducing dysfunction in HRMECs	Wang et al. (2022a)
	Circ-ADAM9	ARPE-19 cells	Up	MiR-338-3p/CARM1	Promoting cell injury	Liang et al. (2022)
	Circ-PSEN1	ARPE-19 cells	Up	MiR-200b-3p/CFL2	Enhancing the ferroptosis of ARPE19 cells	Zhu et al. (2021b)
	CircEhmt1	Vascular pericytes	Up	NFIA/NLRP3	Regulating microvascular dysfunction	Ye et al. (2021)
	Circ_001209	HRMECs	Up	MiR-15b-5p/COL12A1	Increasing diabetic retinal vascular dysfunction	Wang and Zhang, (2021)
	Circ-UBAP2	HRMECs	Up	MiR-589-5p/EGR1	Inducing oxidative stress and dysfunction of HRMECs	Jiewei et al. (2021)
	Hsa_circ_0095008	DR patients	Down	__	Acting as diagnostic biomarkers for the early diagnosis of DR	He et al. (2022)
	Hsa_circ_0001883	DR patients	Up	__	Acting as diagnostic biomarkers for the early diagnosis of DR	He et al. (2022)
DN	Hsa_circ_0001831	DN patients	Up	__	Acting as diagnostic biomarkers for the early type 2 DN	Zhang et al. (2022)
	Hsa_circ_0000867	DN patients	Up	__	Acting as diagnostic biomarkers for the early type 2 DN	Zhang et al. (2022)
	CircEIF4G2	Db/db mouse and NRK-52E cells	Up	MiR-218/SERBP1	Increasing renal fibrosis in DN	Xu et al. (2020)
	CircHIPK3	HK-2 cells	Down	MiR-326, miR-487a-3p/SIRT1	Reducing glucose toxicity to HK-2 Cells	Zhuang et al. (2021)
	Circ_0000712	Kidney tissues and mesangial cells (MCs)	Up	MiR-879-5p/SOX6	Regulating HG-induced apoptosis, inflammation, oxidative stress, and fibrosis in DN	Zhao et al. (2021)
	Circ_0000285	DN mice mouse podocytes	Up	MiR-654-3p/MAPK6	Promoting podocyte injury	Yao et al. (2020)
	CircSMAD4	DN mice and mouse glomerulus mesangial cells (SV40-MES13)	Down	MiR-377-3p/BMP7	Reducing inflammation, extracellular matrix deposition, and apoptosis	Wu et al. (2021b)
	Circ_0037128	DN mice and MCs	Up	MiR-17-3p/AKT3	Promoting the development of DN	Wang et al. (2021c)
	Circ_0000491	DN mice and SV40-MES13 cells	Up	MiR-455-3p/Hmgb1	Promoting apoptosis, inflammation, oxidative stress, and fibrosis	Wang et al. (2021b)
	Circ-AKT3	Mouse DN model and SV40-MES13 cells	Down	MiR-296-3p/E-cadherin	Inhibiting extracellular matrix accumulation	Tang et al. (2020b)
	Circ-FBXW12	Human MCs	Up	MiR-31-5p/LIN28B	Promoting the development of DN	Sun et al. (2021)
	CircRNA_010383	Diabetic kidney of db/db mice	Down	MiR-135a/TRPC1	Contributing to renal fibrosis	Peng et al. (2021)
	CircHIPK3	DN mice and rat MCs	Up	MiR-185/cyclin D1, PCNA, TGF-β1, Col. I, FN	Exacerbating DN and promoting proliferation	Liu et al. (2021b)
	Circ_0080425	Mouse MCs	Down	MiR-24-3p/FGF11	Inhibiting cell proliferation and fibrosis in DN	Liu et al. (2020)
	Circ_15698	Db/db DN mice	Up	MiR-185/TGF-β1	Aggravating accumulation of the extracellular matrix	Hu et al. (2019)
	Hsa_circ_0125310	MCs	Up	MiR-422a/IGF1R, p38	Promoting cell proliferation and fibrosis in DN	Zhu et al. (2022)
	CircTAOK1	Glomerular epithelial cells (GECs)	Up	MiR-520h/SMAD3	Promoting diabetic kidney disease progression	Li et al. (2022)
	Circ-ACTR2	HRMCs	Up	MiR-205-5p/HMGA2	Increasing the dysfunction of human renal MCs	Yun et al. (2021)
	Circ_LARP4	SV40-MES13 cells	Down	MiR-424	Regulating cell proliferation, apoptosis, and fibrosis	Wang et al. (2021d)
	Circ-ITCH	Rat MCs	Down	MiR-33a-5p/SIRT6	Increasing renal inflammation and fibrosis	Liu et al. (2021a)
	Mmu_circRNA_0000309	DN model mice	Down	MiR-188-3p/GPX4	Regulating ferroptosis-depended mitochondrial function and podocyte apoptosis	Jin et al. (2021)
	Circ_0037128	HK-2 cells	Up	MiR-497-5p/NFAT5	Inhibiting cell proliferation and promoting the oxidative stress, inflammation, and fibrosis of HK-2 cells	Wang et al. (2021c)
DNP	CircHIPK3	Serum of DPN patients	Up	MiR-124	Positively associated with grade neuropathic pain in patients with T2D	Wang et al. (2018b)
DCM	CircRNA_000203	Diabetic mouse myocardium	Up	MiR-26b-5p/Col1a2, Col3a1, α-SMA	Enhancing cardiac fibrosis	Tang et al. (2017)
	CircHIPK3	Diabetic mouse myocardium	Up	MiR-26b-5p/Col1a2, Col3a1	Increasing myocardial fibrosis in DCM	Wang et al. (2021e)
	CircHIPK3	AC16 cells	Down	PTEN	Protecting cardiomyocytes from HG-induced cell apoptosis	Jiang et al. (2022)
	CACR	AC16 cells	Up	MiR-214-3p/caspase-1	Regulating pyroptosis in cardiomyocytes	Yang et al. (2019)
	CircRNA_010567	Diabetic mouse myocardium and cardiac fibroblasts	Up	MiR-141/TGF-β1	Promoting myocardial fibrosis	Zhou and Yu, (2017)
	Circ_0071269	H9c2 cells	Up	MicroRNA-145/GSDMA	Promoting the development of DCM	Fu et al. (2022)
Endothelial dysfunction	CircYTHDC2	Vascular smooth muscle cells (VSMCs)	Up	YTHDC2/circYTHDC2/TET2	Promoting the proliferation and migration of VSMCs	Yuan et al. (2021)
	Circ_CLASP2	Human umbilical vein endothelial cells (HUVECs)	Down	MiR-140-5p/FBXW7	Protecting HUVECs from HG-induced dysfunction	Zhang et al. (2021b)
	CircBPTF	HUVECs	Up	MiR-384/LIN28B	Enhancing inflammatory injuries and oxidative stress	Zhang and Sui, (2020)
	CircRNA LRP6	VSMCs	Up	MiR-545-3p/HMGA1	Promoting VSMC proliferation and migration	Bai et al. (2021)
	Hsa_circ_0068087	HUVECs	Up	MiR-197/TLR4, NF-*κ*B, NLRP3	Regulating inflammation and endothelial cell dysfunction	Cheng et al. (2019)
	CircWDR77	VSMCs	Up	MiR-124/FGF2	Promoting VSMC proliferation and migration	Chen et al. (2017)
Wound healing	Circ-Klhl8	Endothelial progenitor cells	Up	MiR-212-3p/SIRT5	Activating autophagy and suppressing endothelial cell damage	Shang et al. (2021)
	CircARHGAP12	Mesenchymal stromal cells	Down	MiR-301b-3p/ATG16L1, ULK2	Activating autophagy and accelerating wound healing	Meng et al. (2021)
	Circ-Gcap14	Adipose-derived stem cells (ADSCs)	Up	MiR-18a-5p/HIF-1α, VEGF	Enhancing angiogenic growth factor expression	Wang et al. (2021f)
	Mmu_circ_0000250	ADSCs	Down	MiR-128-3p/SIRT1	Promoting wound healing in diabetic mice	Shi et al. (2020)

### Diabetic retinopathy

DR is divided into non-proliferative DR and proliferative DR according to severity (Kollias and Ulbig, 2010). DR is a common microvascular complication of diabetes and is the leading cause of blindness in working-aged people (Cheung et al., 2010). It involves changes in pericytes and endothelial cells and is featured by capillary occlusion and vascular leakage (Cheung et al., 2010). CircRNA dysregulation has been implicated in the progression of DR, giving them the potential to act as important biomarkers and rational therapeutics molecules in the future.

#### Biomarker circRNAs

In terms of proliferative DR, circRNA could serve as a diagnostic biomarker. Zhang et al. (Wu et al., 2021a) reported that hsa_circ_0001953 levels were increased in the blood of proliferative DR patients compared with healthy participants and non-proliferative DR patients. However, it needs to verify in a large population. In two diabetic human retinas, it has been found a total of 529 circRNAs were aberrantly expressed. Of these, has_circ_0005015 was verified to be upregulated in the plasma, vitreous sample, and fibrovascular membranes of DR patients, and facilitated retinal endothelial angiogenic function by regulating endothelial cell proliferation, migration, and tube formation. Has_circ_0005015 might be a putative biomarker of DR diagnosis. Further functional experiments found that has_circ_0005015 acts as a miR-519d-3p sponge to inhibit miR-519d-3p activity, leading to increased MMP-2, XIAP, and STAT3 expression (Zhang et al., 2017). The latest research indicated that hsa_circ_0095008 and hsa_circ_0001883, as diagnostic biomarkers for the early diagnosis of DR patients, presented a diagnostic capability with AUCs of 0.671 and 0.607, respectively (He et al., 2022).

#### Therapeutic circRNAs

CircHIPK3 expression was up in diabetic retinas and retinal endothelial cells. It increased vascular endothelial growth factor-C, FZD4, and WNT2 expression by blocking miR-30a-3p, which led to the endothelial proliferation and vascular dysfunction (Shan et al., 2017). Moreover, studies found that circDNMT3B, circFTO, circ_001209, circCOL1A2, circ-UBAP2, circSLC16A12 and exosomal circFndc3 can also regulate diabetic retinal vascular function (Zou et al., 2020; Guo et al., 2021; Jiewei et al., 2021; Li et al., 2021; Wang et al., 2022a). All of these are potential targets to control DR. CircDNMT3B downregulation exacerbated visual damage and circDNMT3B overexpression contributed to the alleviation of retinal vascular dysfunction, while circ_001209 upregulation aggravated the retinal injury by sponging miR-15b-5p in diabetic rats (Zhu et al., 2019; Wang and Zhang, 2021). In addition, circRNAs can regulate DR by influencing the crosstalk between pericytes and endothelial cells (Liu et al., 2019; Ye et al., 2021). A novel DR-associated circRNA cPWWP2A acted as a competing endogenous RNA by interacting with miR-579 to promote retinal vascular dysfunction through the up-regulation of angiopoietin 1, occludin, and SIRT1 (Liu et al., 2019). What’s more, circRNA cPWWP2A exists in pericytes but not in endothelial cells, but it can indirectly regulate the biology of endothelial cells through exosomes. Exosomal circEhmt1 released from pericytes can be transferred from pericytes to endothelial cells, and upregulated circEhmt1 protects endothelial cells against injury in the high glucose condition (Ye et al., 2021). CircRNAs also play an important role in human retinal pigment epithelial (ARPE 19) cells. Studies showed that hsa_circ_0041795 and circRNA_0084043 were obviously elevated in HG-induced ARPE-19 cells, attenuating ARPE-19 cell proliferation and survival. Mechanically, hsa_circ_0041795 contributed to HG-treated ARPE-19 cell apoptosis by mediating the activation of VEGFC *via* sponging miR-646 (Sun and Kang, 2020). Loss of circRNA_0084043 significantly increased HG-triggered apoptosis of ARPE-19 cells by sponging miR-140-3p and inducing TGFA. Meanwhile, deficiency of circRNA _0084043 effectively suppressed HG-stimulated inflammation by inhibiting the inflammatory cytokines TNF-α, IL-6, and Cox-2 in ARPE-19 cells (Li et al., 2020b). Circ-ADAM9 and circ-PSEN1 were also up-regulated in DR, circ-ADAM9 could promote ARPE-19 cell injury and downregulation of circ-PSEN1 can ameliorate ferroptosis (Zhu et al., 2021b; Liang et al., 2022). CircZNF532 not only regulates pericyte biology by acting as a miR-29a-3p sponge and inducing increased expression of NG2, LOXL2, and CDK2, but also modulates ARPE-19 cell apoptosis and pyroptosis, thereby facilitating angiogenesis and inflammation in DR (Jiang et al., 2020; Liang et al., 2021b; Wang et al., 2022b). In rat retinal pigment epithelial cells, it has been found circ-ITCH involved in the physiology and pathology of DR (Zhou et al., 2021). The latest research showed that the silence of circMET can reduce retinal angiogenesis (Yao et al., 2022). Altogether, circRNA was not only involved in the progression of DR but also exhibited a promising therapeutic potential in the treatment of DR.

### Diabetic nephropathy

DN is a common cause of death in diabetes patients (Tuttle et al., 2014). Pathologic features of DN include the progressive accumulation of the extracellular matrix (ECM), the continuous proliferation of mesangial cells (MCs), the thickening of the basement membrane, and kidney fibrosis (Wang et al., 2018a). ECM and MC dysfunction are crucial players and early warning signs in the pathogenesis of DN. Unraveling the molecular mechanisms behind DN and identifying promising therapeutic targets can lead to the improved survival of patients with complications from diabetes.

#### Biomarker circRNAs

Currently, several studies have reported that circRNAs as biomarkers of DR. Hsa_circ_0001831 and hsa_circ_0000867 were upregulated in the peripheral blood of early type 2 DN patients (Zhang et al., 2022). It was found that hsa_circ_0001831 has a good diagnostic value for early type 2 DN with a sensitivity of 0.85, a specificity of 0.85, and an AUC value of 0.95 (Zhang et al., 2022). Additionally, in a study including 73 patients, the result showed that hsa_circRNA_102682 might be an independent risk factor of DR. The ROC curve demonstrated that the AUC of hsa_circRNA_102682 is 0.97 (Hu et al., 2021).

#### Therapeutic circRNAs

Numerous reports have highlighted circRNAs in the treatment of DN, such as circEIF4G2, hsa_circ_0125310, circTAOK1, and circ-ACTR2 (Xu et al., 2020; Yun et al., 2021; Li et al., 2022; Zhu et al., 2022). A study has indicated that circ-FBXW12 was increased in the serum of DN patients and human MCs by HG-induced. Functional experiments *in vitro* showed that circ-FBXW12 silencing suppressed human MC growth and reduced ECM production and oxidative stress by regulating the miR-31-5p/LIN28B pathway (Sun et al., 2021). CircSMAD4 could alleviate ECM deposition, while circRNA_15698 could aggravate ECM deposition (Hu et al., 2019; Wu et al., 2021b). Liu et al. (Liu et al., 2020) demonstrated that the expression of circ_0080425 correlates with DN progression and has a positive effect on cell proliferation and fibrosis in MCs *via* the miR-24-3p/FGF11 signaling axis. Circ_0000712 potentiated HG-caused apoptosis, inflammation, oxidative stress, and fibrosis in DN by targeting the miR-879-5p and regulating SOX6 expression (Zhao et al., 2021). Similarly, circ_0000491 could regulate Hmgb1 expression by sponging miR-455-3p, thereby exacerbating HG-induced apoptosis, inflammation, oxidative stress, and fibrosis in DN (Wang et al., 2021b). *In vivo*, overexpression of circRNA_010383 downregulated miR-135a function, upregulated transient receptor potential cation channel, subfamily C, and member 1 levels, and inhibited proteinuria and renal fibrosis in DN mice (Peng et al., 2021). Further, circ_0000285 and circ_0037128 were increased in mice models of DN. Circ_0000285 can serve as a sponge for miR-654-3p, activates the expression levels of MAPK6, and results in podocyte injury (Yao et al., 2020). Recent evidence has shown that circ_0037128 regulates the development of DN *via* the miR-17-3p/AKT3 pathway (Wang et al., 2021c). An additional recent work found that circ_0037128 inhibits cell proliferation and promotes the inflammation and fibrosis of HK-2 cells through the miR-497-5p/NFAT5 axis. In contrast, the levels of circ-AKT3, circ_LARP4, circ-ITCH, and mmu_circRNA_0000309 were downregulated in mice or rats with DN or in DN tissue or cell models (Tang et al., 2020b; Liu et al., 2021a; Wang et al., 2021d; Jin et al., 2021). Upregulation of circ-AKT3 can inhibit the synthesis of fibrosis-associated protein through sponging miR-296-3p to modulate E-cadherin, which might provide a non-negligible potential opportunity for therapeutic targets of patients with DN (Tang et al., 2020b). CircHIPK3, as mentioned above, regulates islet proliferation and insulin secretion, thereby contributing to insulin resistance. Two studies on DN reported that circHIPK3 has a major influence on human renal tubular epithelial HK-2 cells and rat MCs. Mechanistically, circHIPK3 expression was remarkably downregulated in HK-2 cells that were HG-treated. The overexpression of circHIPK3 reduced miR-326 and miR-487a-3p accumulation, leading to the upregulation of SIRT1 and reduced HG-induced inhibition of HK-2 proliferation (Zhuang et al., 2021). While another study showed that the expression levels of circHIPK3 in rat MCs were increased. Knocking down circHIPK3 inhibited cell proliferation and decreased the cyclin D1, PCNA, TGF-β1, Col. I, and FN mRNA abundance in MCs (Liu et al., 2021b). Additional studies are needed to confirm the role of circHIPK3 in DN.

#### Diabetic neuropathy

DNP is one of the most prevalent diabetic complications and significantly decreases the quality of life of diabatic patients. DNP is a loss of sensory function that begins in the distal lower extremities that are also characterized by spontaneous and stimulus-evoked pain and numerous morbidities (Feldman et al., 2019). However, the etiology behind DNP remains unclear. Several works have shown that circRNAs play an important regulatory role in the progression of DNP.

#### Biomarker circRNAs

Recent works have evaluated the expression of noncoding RNAs, includingmiRNAs, long ncRNAs, and circRNAs in streptozotocin-induced DNP mice. A total of 30 mRNAs, 148 miRNAs, 9 long ncRNAs, and 135 circRNAs exhibited significantly dysregulated expression in DNP mice. These factors involved the protein digestion and absorption pathways, the Rap1 signaling pathway, human TT-lymphotropic virus-I infection, and the MAPK signaling pathway (He et al., 2021). Another study identified 15 circRNAs and 133 mRNAs with altered expression levels in the dorsal root ganglia of wild-type and diabetic mice using high-throughput RNA sequencing and a chip scan. 7 circRNAs and 14 mRNAs were significantly correlated with diabetic peripheral neuropathy (Zhang et al., 2020b). These circRNAs may serve as biomarkers for the prediction and diagnosis of DNP in the future.

#### Therapeutic circRNAs

It has been demonstrated that circHIPK3 was abundant in DNP patients and rats. Upregulation of circHIPK3 was positively associated with neuropathic pain in patients with T2D. Silencing circHIPK3 alleviated neuropathic pain in diabetic rats, which was attributed to neuroinflammation. A mechanistic investigation found that circHIPK3 interacted with miR-124 and down-regulated its expression. This is the first evidence that intrathecal circHIPK3 shRNA treatment can be used to treat DNP rats (Wang et al., 2018b).

### Diabetic cardiomyopathy

DCM is a major cause of morbidity and mortality in the diabetic population as well as a heavy burden on social economies worldwide. DCM is characterized by cardiomyocyte hypertrophy, apoptosis, myocardial interstitial fibrosis, and later heart failure in the absence of dyslipidemia, coronary artery disease, and hypertension (Jia et al., 2018; Dillmann, 2019).

CircRNAs have vital effects on the progression of DCM. Zhou and coworkers indicated that circRNA_010567 in diabetic mice myocardium and Ang- II-induced cardiac fibroblasts (CFs) significantly increased and modulated the excision of fibrosis-associated proteins in CFs, including Col I, Col III, and α-SMA *via* the miR-141/TGF-β1 signaling axis (Zhou and Yu, 2017). CircRNA_000203 levels were markedly elevated in the DCM mouse myocardium and CFs. Moreover, functional experiments found that overexpression of circRNA_000203 suppresses miR-26b-5p and activates Col1a2 and CTGF, ultimately resulting in the increased expressions of Col3a1 and α-SMA in CFs (Tang et al., 2017). CircHIPK3 was later found to contribute to enhanced myocardial fibrosis and reduced cardiac function in DCM mice, and loss of circHIPK3 inhibits the proliferation of CFs *via* the miR-29b-3p/Col1a1, Col3a1 regulatory network (Wang et al., 2021e). These results indicate that circRNA_010567, circRNA_000203, and circHIPK3 are involved in the pathogenesis of myocardial fibrosis, which can provide a molecular mechanism and therapeutic target for DCM.

Another study pointed out that circHIPK3 was downregulated in DCM and HG-treated human cardiac myocytes. Overexpression of circHIPK3 suppressed the expression levels of PTEN, which is a kind of tumor suppressor gene, and inhibited human cardiac myocytes apoptosis treatment by HG (Jiang et al., 2022). Circ_0071269 was significantly accumulated in rat cardiomyocytes after treatment with HG. Knockdown of circ_0071269 promoted cell viability and inhibited the inflammatory response, cytotoxicity, and pyroptosis of rat cardiomyocytes *in vitro* by sponging miR-145 to upregulate GSDMA (Fu et al., 2022). Hsa_circ_0076631, also known as caspase-1-associated circRNA (CACR), was increased in both diabetic patients and cardiomyocytes treated with HG. Knocking down CACR-activated caspase-1 *via* miR-214-3p in cardiomyocytes. Conversely, the loss of miR-214-3p partially abolished the beneficial effects of CACR silencing on pyroptosis in cardiomyocytes. Therefore, this study concluded that CACR might be a novel therapeutic target for modulating the CACR/miR-214-3p/caspase-1 pathway in DCM (Yang et al., 2019).

### Hyperglycemia-related endothelial dysfunction and wound healing

Endothelial cells comprise the inner lining of blood vessels and act as an anticoagulant barrier between the vessel wall and the blood (Sumpio et al., 2002). Endothelial cells are unique multifunctional cells that are charactered by the regulation of metabolic hemostasis, vasomotor tone, vascular smooth muscle cell (VSMC) proliferation, and an immune and inflammatory response (Kaur et al., 2018b). Previous studies have shown that hyperglycemia can lead to damaged endothelial cell function in diabetes patients (Ceriello, 2010; Grassi et al., 2012). Accumulated data indicates that hyperglycemia profoundly alters circRNAs expression and circRNAs are involved in the diverse biological process of HG-induced endothelial dysfunction in diabetes (Shang et al., 2018).

Human umbilical vein endothelial cells (HUVECs) are readily available and widely used for *in vitro* studies of endothelial cells. Cheng et al. (Cheng et al., 2019) reported that hsa_circ_0068087 promoted inflammation and endothelial cell dysfunction by activating the miR-197/TLR4/NF-*κ*B/NLRP3 pathway. Another, circBPTF was highly regulated in HG-induced HUVECs. Loss of circBPTF inhibited inflammatory injuries and oxidative stress by targeting miR-384 and downregulating the expression of LIN28B (Zhang and Sui, 2020). In contrast, it has been demonstrated that circ_CLASP2 is decreased in HG-induced HUVECs. Circ_CLASP2 protects HUVECs against hyperglycemia dysfunction by regulating the miR-140-5p/FBXW7 pathway (Zhang et al., 2021b).

Significant evidence has suggested that circRNAs such as circWDR77, circYTHDC2, and circLRP6 are highly expressed in VSMCs treated with HG (Chen et al., 2017; Bai et al., 2021; Yuan et al., 2021). All of these circRNAs promote the proliferation and migration of VSMCs through different mechanisms. Silencing circWDR77 suppresses VSMC proliferation and migration by targeting the miR-124/fibroblast growth factor 2 signaling axis (Chen et al., 2017). CircYTHDC2 regulates the progression of VSMC dysfunction *via* YTHDC2-mediated m6A modification and by modulating the expression of ten-eleven translocation 2 (Yuan et al., 2021). CircLRP6, a miR-545-3p sponge, facilitates the proliferation and migration of HG-induced VSMCs by decreasing HMGA1 expression (Bai et al., 2021).

Several studies found that different circRNAs are involved in hyperglycemia-related wound healing. Circ-Klhl8 levels in endothelial progenitor cells and circ-Gcap14 levels in adipose-derived stem cells (ADSCs) are upregulated in the setting of hypoxic pretreatment conditions (Wang et al., 2021f; Shang et al., 2021). Overexpression of circ-Gcap14 promotes angiopoiesis and accelerates hypoxic ADSC-mediated diabetic wound closure (Wang et al., 2021f). Shang et al. (Shang et al., 2021) reported that circ-Klhl8 increases autophagy, inhibits HG-induced endothelial cell damage, and promotes diabetic wound healing by regulating the miR-212-3p/SIRT5 signaling axis. Recent studies have reported that both exosomal mmu_circ_0000250 and circARHGAP12 have the capability to accelerate diabetic wound healing by mediating autophagy (Shi et al., 2020; Meng et al., 2021). Mechanistically, circARHGAP12 promotes diabetic wound closure by sponging miR-301b-3p to regulate the expression of the target genes ATG16L1 and ULK2 (Meng et al., 2021). CircARHGAP12 may be a new target for the treatment of diabetic wound healing.

## Conclusion and perspectives

With the development and progress of science and technology, new research perspectives related to circRNAs have attracted significant attention. First, the association of circRNAs with exosomes and m6A methylation has become a hot topic. CircRNAs can be encapsulated in exosomes and delivered out of cells to serve as regulators for cell-to-cell communication (Wang et al., 2019). M6A methylation modification not only can regulate circRNA translation but also facilitate circRNA degradation. Increasing evidence indicated that m6A-modified circRNAs play an important role in innate immunity and tumors (Zhang et al., 2020c). It has been demonstrated that m6A modification of circNSUN2 enhances HMGA2 stability and modulates the progression of colorectal liver metastasis by facilitating cytoplasmic export and forming a circNSUN2/IGF2BP2/HMGA2 RNA-protein ternary complex (Chen et al., 2019). Second, given the universality, stability, and diversity of circRNA, it can sever as a promising biomarker for various diseases and has been proposed to be applied to conduct vaccine development. The latest study reported that a circRNA vaccine has a durable ability to produce antigens, neutralize antibodies, and protect against SARS-CoV-2. It produces its effect by encoding the RNA-binding domain of the spike protein (Qu et al., 2022). Last, nanoparticles-based carrying and delivery of therapeutic agents targeting circRNAs in kinds of diseases are emerging. Recently, Du et al. (Du et al., 2022) utilized a siRNA vector poly (β-amino esters) (PAEs) targeting circMDK to develop a PAE-siRNA complex delivery. The PAE-siRNA complex could downregulate circMDK and inhibit hepatocellular carcinoma without obvious side effects in tumor models. Additionally, *in vitro* transcribed circ-INSR mimics effectively prevented doxorubicin-induced cardiotoxicity, which provides an exciting complementary option for adeno-associated virus-based therapies (Lu et al., 2022).

In summary, significant evidence has identified roles for circRNAs in a variety of cells, organs, and diseases including pancreatic islet cells, different subtypes of diabetes, and diabetic complications. In-depth studies on the biologic pathogenesis and precise molecular function of circRNAs in diabetes are of great scientific significance and potential clinical application. Considering the regulatory roles played by circRNAs in islet cells, immune cells, and other diabetic complication organ-specific tissue cells, we believe that circRNAs can be used as an effective strategy for treating diabetes and its complications. Once the precise molecular mechanisms and functions of circRNAs are understood, more surprises and breakthroughs for the prevention, diagnosis and treatment of diabetes are expected.
